# ECMO attenuates inflammation response and increases ATPase activity in brain of swine model with cardiac arrest compared with CCPR

**DOI:** 10.1042/BSR20182463

**Published:** 2019-07-19

**Authors:** Yun Zhang, Chun-Sheng Li, Xiao-Li Yuan, Ji-Yang Ling, Qiang Zhang, Yong Liang, Bo Liu, Lian-Xing Zhao

**Affiliations:** 1 Department of Emergency Medicine, Beijing Tongren Hospital, Capital Medical University, Beijing, China; 2 Department of Emergency Medicine, Beijing Chaoyang Hospital, Capital Medical University, Beijing, China

**Keywords:** ATPase activity, Brain, cardiac arrest, extracorporeal membrane oxygenation, inflammation

## Abstract

Extracorporeal membrane oxygenation (ECMO) could increase survival rate and neurological outcomes of cardiac arrest (CA) patients compared with conventional cardiopulmonary resuscitation (CCPR). Currently, the underlying mechanisms how ECMO improves neurological outcomes of CA patients compared with CCPR have not been revealed. A pig model of CA was established by ventricular fibrillation induction and then underwent CCPR or ECMO. Survival and hemodynamics during the 6 h after return of spontaneous circulation (ROSC) were compared. The levels of inflammatory cytokines and Ca^2+^-ATPase and NA^+^-K^+^-ATPase activities were detected. Brain tissues histology and ultra-microstructure in CCPR and ECMO groups were also examined. Results suggested that ECMO significantly improved the survival of pigs compared with CCPR. Heart rate (HR) decreased while cardiac output (CO) increased along with the time after ROSC in both ECMO and CCPR groups. At each time point, HR in ECMO groups was lower than that in CCPR group while CO and mean arterial pressure in ECMO group was higher than CCPR group. In ECMO group, lower levels of IL-1, IL-1β, IL-6, TNFα, and TGFβ, especially IL-1, IL-6, TNFα, and TGFβ, were found compared that in CCPR group while no difference of IL-10 between the two groups was observed. Similar with the results from enzyme-linked immunosorbent assay, decreased expressions of IL-6 and TGFβ were also identified by Western blotting. And Ca^2+^-ATPase and NA^+^-K^+^-ATPase activities were increased by ECMO compared with CCPR. Hematoxylin and eosin staining and ultra-microstructure examination also revealed an improved inflammation situation in ECMO group compared with CCPR group.

## Introduction

Cardiac arrest (CA), with very poor survival and neurological outcome, is the leading cause of sudden death and accounts for appropriate half of all cardiac deaths [1–5]. In past decades, extracorporeal membrane oxygenation (ECMO), also called extracorporeal life support (ECLS), has been used as an alternative resuscitative method for patients with refractory CA in the emergency department, intensive care unit, or catheterization room [6,7]. A series of recent studies suggested that, compared with conventional cardiopulmonary resuscitation (CCPR), extracorporeal cardiopulmonary resuscitation (ECPR) with ECMO strategies may improve survival and neurological outcomes in both out of hospital CA (OHCA) and in of hospital CA (IHCA), especially in IHCA [8–12]. Survival to discharge rates of ECPR have been reported to be 4–36% for adult OHCA and 34–46% for adult IHCA [13,14], while survival to discharge rates following CCPR have been estimated at 10–20% for CA [5,15–17]. A recent meta-analysis suggested that the use of ECMO is associated with an absolute increase of 30-day survival of 13% compared with patients not receiving ECMO and a higher rate of favorable neurological outcome at 30-days [18].

Unfortunately, the underling mechanisms that ECMO is associated with more favorable neurological outcomes compared with CCPR in CA patients are not clear. Animal experiments suggest that inflammatory cytokines expressions such as IL-1β, IL-6, IL-8, IL-10, and TNFα are aberrantly expressed in blood as well as in tissues after CA and cardiopulmonary resuscitation (CPR) [19]. TNFα, IL-1β, and IL-6 up-regulation in brain can lead to nerve cell apoptosis and necrosis [20], glial cells, neurons, axonal injury, and brain tissue edema [21], neurotransmitter transporter obstacles [22], the destruction of the blood–brain barrier (BBB) [23] and increased oxidative stress injury of the brain tissue [24,25], while IL-10 has a significant protective effects on brain injury [26]. Thus, we hypothesized that ECMO exerts protective effects on brain via attenuating inflammation in brain and then contributes to a high survival rate and favorable neurological outcomes. However, currently, the brain inflammation in CA after ECMO and CCPR has not been investigated.

In the present study, we established a pig model of CA and then compared the inflammatory cytokines levels in brain tissues after CCPR or ECMO treatment. In addition, the ATPase activities were also compared.

## Materials and methods

### Animals and experimental protocol

The present study was conducted in accordance with the guideline for the Animal Care and Use, established by the Beijing Chaoyang Hospital of the Capital Medical University, and all experimental protocols were approved by the Ethics Committee of Animal Experiments of Capital Medical University.

Sixteen Chinese landrace pigs (27.0 ± 2.5 kg; 12–14 weeks) were fasted overnight with free access to water. Then, the pigs were pre-anesthetized with 0.5 mg/kg midazolam intra-muscular injection and 1.0 mg/kg continuous infusion in ear vein. Next, the pigs were placed securely on the anesthesia table and their sedation was maintained with an intravenous infusion of pentobartibal (8 mg/kg/h). After that, endotracheal intubation into trachea and conventional mechanical ventilation with a volume-controlled ventilator (Drager vi 400, Munich, Germany) were performed. The following parameters were monitored: tidal volume, 15 ml/kg; fraction of inspired oxygen (FiO_2_), 0.21; and respiratory frequency, 12 breaths/min. The end-tidal partial pressure of carbon dioxide (PCO_2_) was measured by an inline infrared capnography (CO2SMO plus monitor; Respirometric Inc, Murrysville, PA). Room temperature was maintained at 26°C and core temperature of the animals was maintained at 37.5 ± 0.5°C. And then, they were instrumented with a carotid output flow probe (PS-Series Probes, Transonic, NY), two fluid-filled catheters into the left carotid artery and right atrium, and a hand-made pneumatic occluder around the proximal left descending coronary artery. Two cannulae were also inserted into the right femoral vessels, one arterial (15F) and one venous (19F; HLS; Maquet, Rastatt, Germany) for further implementation of ECPR. During the experiments, carotid blood flow, arterial blood pressure, and electrocardiogram (ECG) were continuously recorded. Arterial blood samples were collected at baseline every 60 min after CCPR or ECMO initiation.

The experimental protocols were as follows: after instrumentation, the pigs were equilibrated for 30 min to achieve stabilization. Next ventricular fibrillation was induced by programmed electric stimulation (30 V) and confirmed by the VF wave in the ECG. Then conventional CPR was performed in all pigs by continuous external chest compressions using an automated device (100 compressions/min, Shanghai Henlong Science and Education Equipment LTD) and asynchronous ventilation. The compression-to-ventilation ratio was 30:2. After 10 min of conventional CPR, the pigs were randomly divided into CCPR group (*n*=8) and ECMO group (*n*=8). The pigs of CCPR group continued to receive CCPR for 360 min, while the pigs of ECMO group were shifted to VA-ECMO for 360 min using a transportable device (Cardiohelp; Maquet). Accordingly, circuit and membrane oxygenator (HLS; Maquet) were primed and heparinized (5000 IU). Extracorporeal blood flow was progressively increased to 50–60 ml/kg/min to optimize the blood flow of the animals in order to make them to achieve a highest mean arterial pressure (MAP). The research endpoints were death of the animals or 6 h after return of spontaneous circulation (ROSC). The ROSC was defined as MAP was beyond 60 mmHg for 20 min or a systolic blood pressure was over 80 mmHg. CPR in CCPR groups was stopped after ROSC. When ROSC was achieved, the time was set to 0 h. Blood samples were collected for laboratory testing at 0, 1, 2, 4, and 6 h after ROSC (ROSC 0 h, ROSC 1 h, ROSC 2 h, ROSC 4 h, and ROSC 6 h, respectively) or until the animals died. At the end of the research, the surviving animals were killed and the frontal cortex samples of the left hemisphere of brain, which were closely rated to the functional outcomes, were rapidly harvested, snap-frozen in liquid nitrogen immediately, and subsequently stored at −80°C for further study.

#### Hemodynamics

Hemodynamic parameters, including heart rate (HR), MAP, and cardiac output (CO), were monitored. HR was recorded by the standard lead II ECG. MAP was measured with a fluid-filled catheter advanced from the left femoral artery into the aortic arch through a pressure transducer with pulse indicator continuous COCO system. A Swan–Ganz catheter (7 Fr; Edwards Life Sciences, Irvine, CA, U.S.A.) was advanced from the left femoral vein and flow directed into the pulmonary artery to measure CO by the thermodilution method. All the values were recorded at baseline, at ROSC 0 h (ROSC0), and at 1 h (ROSC1), 2 h (ROSC2), 4 h (ROSC4), and 6 h (ROSC6) after ROSC.

### Detection of IL-1, IL-1β, IL-6, IL-10, TNFα, TGFβ, and KL-6 levels in brain

Frozen cerebral cortex samples were firstly homogenized in cold-PBS supplied with protease inhibitors (Sigma–Aldrich, St Louis, MO, U.S.A.). The sample weight to PBS ratio was 1 g: 9 ml. To further break the cells, the samples were sonicated with an ultrasonic cell disrupter. Then the homogenates were contrifugated for 5 min at 5000 × *g* to get the supernatants. The concentration of the total proteins was determined by the Bradford method. The levels of IL-1, IL-1β, IL-6, IL-10, TNFα, TGFβ, and KL-6 were detected via an enzyme-linked immunosorbent assay (ELISA) according to the manufacturer’s instructions (Shanghai Renjie Biotech LTD). Each sample was detected in triplicate. The proteins expression levels of IL-1β, IL-6 (Abcam, U.S.A.), IL-10 (Sangon, Shanghai, China), and TGFβ (R&D Systems, U.S.A.) were also examined by Western blotting with corresponding primary antibodies and secondary antibodies. The optical densities of the immunoreactive bands were assessed using Image J software. The protein levels were normalized to Tubulin and expressed as ratios.

### Ca^2+^-ATPase and NA^+^-K^+^-ATPase activity detection

Ca^2+^-ATPase and NA^+^-K^+^-ATPase activities were measured as ATP cleavage in a medium containing (mmol/l) 130 NaCl, 20 KCl, 3 MgCl_2_, 3 ATP, and 30 imidazole, pH 7.4, at 37°C. Inorganic phosphate concentration was determined with colorimetric assays according to the manufacturer’s instructions.

### Histology and ultramicrostructure examination

Brain tissues were fixed with 10% formalin, dehydrated and embedded in paraffin, cut into section, stained with hematoxylin and eosin, and observed by an optical microscopy (CX41; Olympus, Tokyo, Japan). For ultra-microstructure examination, brain tissues were cut into pieces (1.0 mm × 1.0 mm × 1.0 mm), treated with 3% glutaraldehyde, flushed with phosphate-buffered saline, fixed with 1% perosmic acid, and dehydrated with acetone. Ultrathin sections were placed on 200-mesh copper grids and double stained with 4% uranyl acetate and 0.2% lead citrate. Sections were examined under transmission electron microscopy (H-7650; Hitachi, Ibaraki, Japan).

### Statistical analysis

Data were expressed as mean ± standard deviation (SD) for continuous variables. Survival after ROSC of the animals between CCPR and ECMO groups were compared with Kaplan–Meier plot curves and Log-Rank test. Hemodynamic parameters between CCPR and ECMO groups were compared with two-way ANOVA and Sidak’s multiple comparisons test. The difference of the other variables between CCPR and ECMO groups was compared by unpaired Student *t* test. Nonparametric data was expressed as median (interquartile range) and analyzed by the Mann–Whitney U test. All statistical analysis was performed with GraphPad Prism 6.0 software (GraphPad Software, Inc. CA, U.S.A.). A two-tailed *P*<0.05 was considered as statistical significance.

## Results

### Survival

**Figure 1:**
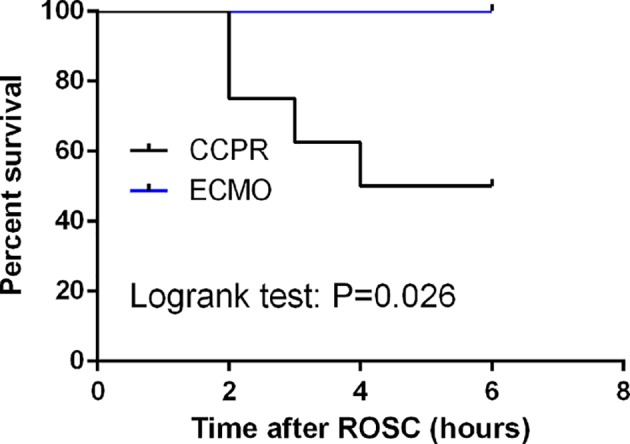
Comparison of survivals rate and rescue time for ROSC in CA swine undergoing EPCR (*n*=8) or CCPR (*n*=8) CCPR, conventional cardiopulmonary resuscitation; ECMO, extracorporeal membrane oxygenation; ECPR, extracorporeal cardiopulmonary resuscitation with ECMO strategies; ROSC, return of spontaneous circulation.

All the animals in both ECMO and CCPR groups achieved ROSC. During 6 h-follow up after ROSC, the following 6 h observation after ROSC, 100% (8/8) swine survived in ECMO groups while 50% (4/8) swine survived in CCPR group (Figure 1).

### Hemodynamics

**Figure 2:**
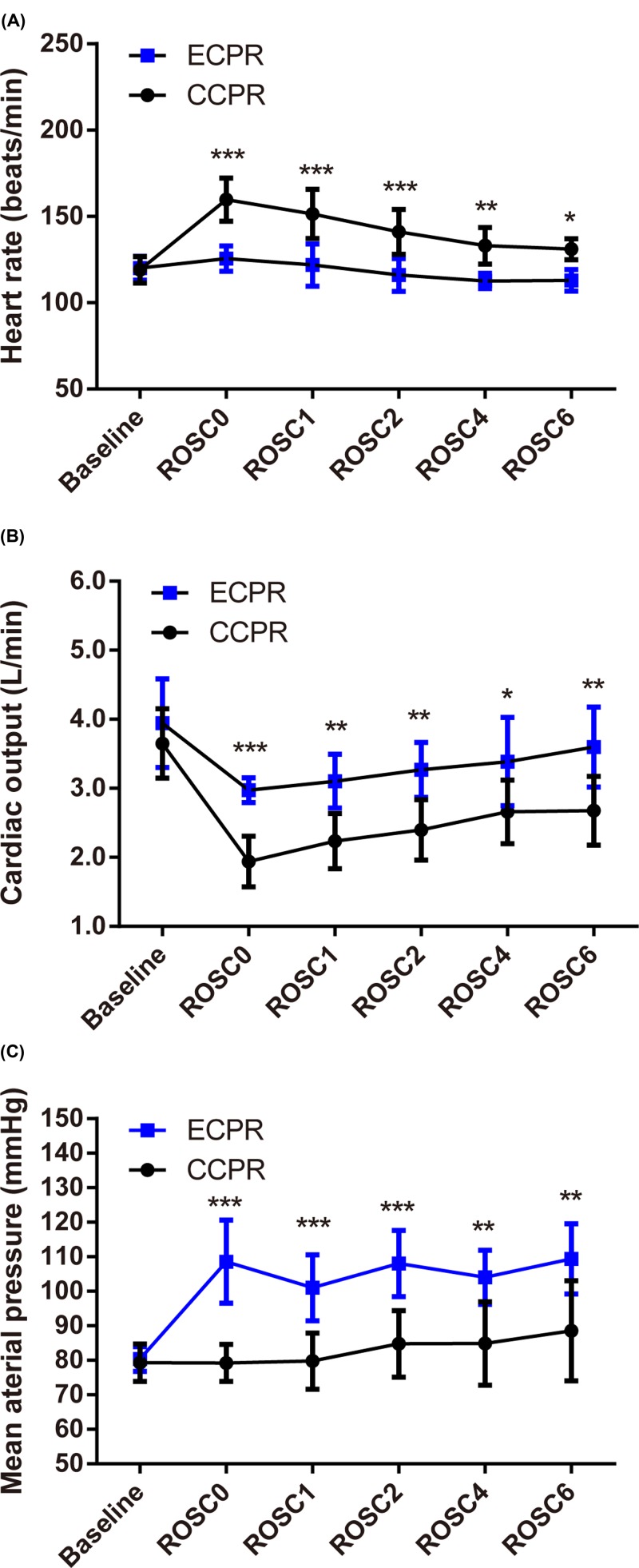
Comparison of hemodynamics in CA swine undergoing ECMO (*n*=8) or CCPR (*n*=8) (**A**) HR; (**B**) CO; (**C**) MAP. *P<0.05; **P<0.01; ***P<0.001; CCPR, conventional cardiopulmonary resuscitation; ECMO, extracorporeal membrane oxygenation; ECPR, extracorporeal cardiopulmonary resuscitation with ECMO strategies; ROSC, return of spontaneous circulation.

HR, CO, and MAP at baseline between ECMO group and CCPR group were comparable. HR decreased while CO increased along with the time after ROSC in both ECMO and CCPR groups (Figure 2A,B). At each time point, HR in ECMO groups was lower than that in CCPR group, while CO in ECMO group was higher than CCPR group (Figure 2A,B). In addition, MAP was higher in ECMO group compared with CCPR group (Figure 2C).

### Inflammation in pig brain was relieved by ECMO after ventricular fibrillation

**Figure 3:**
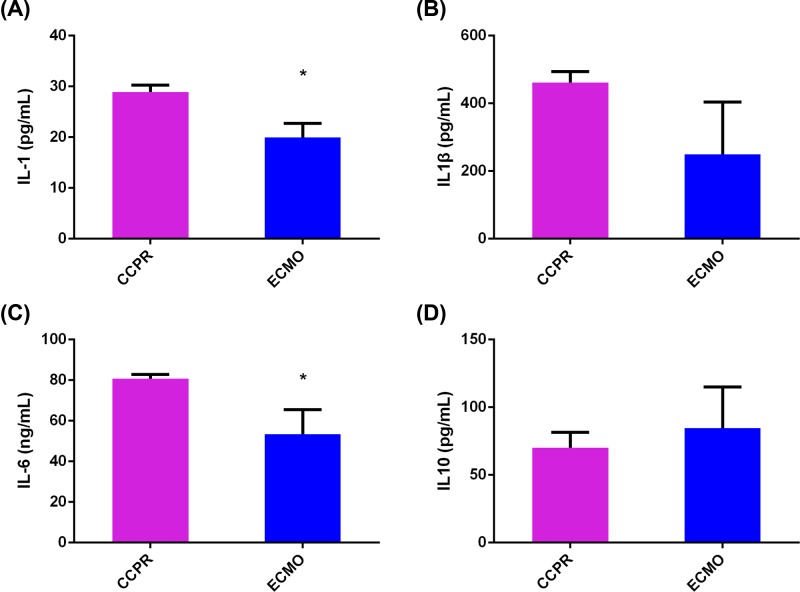
ELISA detection of IL-1, IL-1β, IL-6, and IL-10 in brain tissues of pigs with ventricular fibrillation undergoing EPCR (*n*=8) or CCPR (*n*=4) (**A**) IL-1, (**B**) IL-1β, (**C**) IL-6, and (**D**) IL-10. *P<0.05; CCPR: Conventional cardiopulmonary resuscitation; ECMO: Extracorporeal membrane oxygenation; ECPR: Extracorporeal cardiopulmonary resuscitation with ECMO strategies; ROSC: Return of spontaneous circulation.

**Figure 4:**
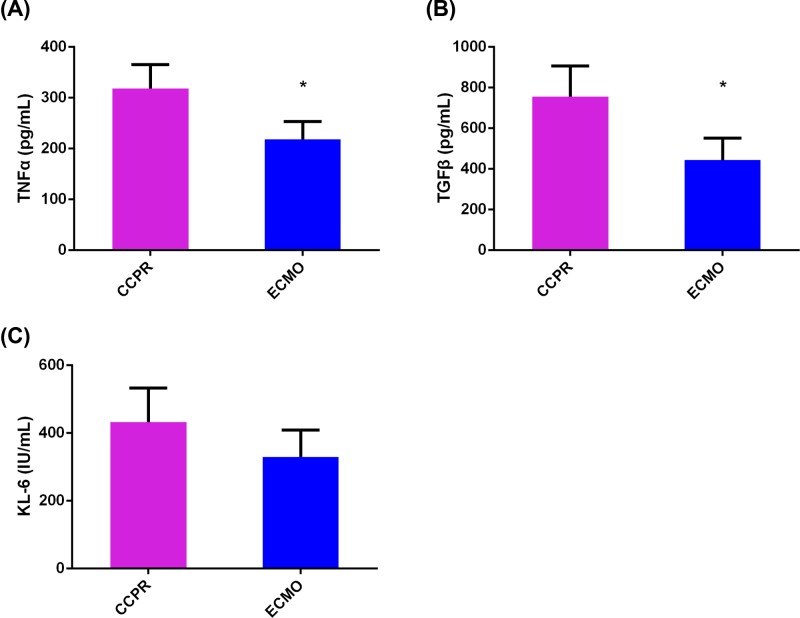
ELISA detection of TNFα, TGFβ, and KL-6 in brain tissues of pigs with ventricular fibrillation undergoing EPCR (*n*=8) or CCPR (*n*=4) (**A**) TNFα, (**B**) TGFβ, and (**C**) KL-6. *P<0.05; CCPR, conventional cardiopulmonary resuscitation; ECMO: Extracorporeal membrane oxygenation; ECPR, extracorporeal cardiopulmonary resuscitation with ECMO strategies; ROSC, return of spontaneous circulation.

**Figure 5:**
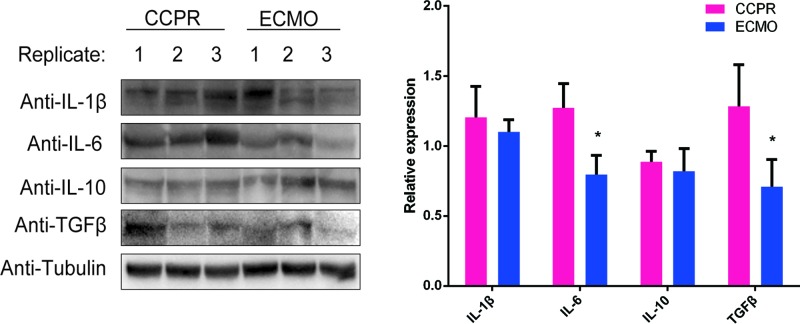
Western blotting analysis of IL-1β, IL-6, IL-10, and TGFβ in brain tissues of pigs with ventricular fibrillation undergoing EPCR (*n*=8) or CCPR (*n*=4) IL-1β, IL-6, IL-10 and TGFβ. *P<0.05; CCPR, conventional cardiopulmonary resuscitation; ECMO, extracorporeal membrane oxygenation; ECPR, extracorporeal cardiopulmonary resuscitation with ECMO strategies; ROSC, return of spontaneous circulation.

The cerebral cortex samples of the pigs were homogenized and sonicated, and the inflammation cytokines in the samples were determined by ELISA. In ECMO group, the levels of IL-1, IL-1β, IL-6, TNFα, and TGFβ (especially IL-1, IL-6, TNFα, and TGFβ) were lower compared with CCPR group (Figures 3A–C and 4A,B), while no difference of IL-10 levels between the two groups was observed (Figure 3D). In addition, KL-6 was also examined and only a trend of decrease in ECMO group was identified (Figure 4C). Then the protein expression levels of IL-1β, IL-6, IL-10, and TGFβ were further validated by Western blotting. Similar with the results from ELISA, decreased of IL-6 and TGFβ in ECMO group were also identified (Figure 5).

### ECMO increase ATPase activity in pig brain after ventricular fibrillation

**Figure 6:**
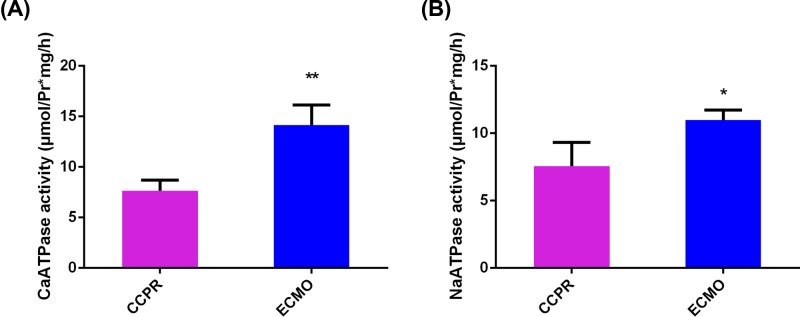
Detection of Ca^2+^-ATPase and NA^+^-K^+^-ATPase activity in brain tissues of pigs with ventricular fibrillation undergoing EPCR (*n*=8) or CCPR (*n*=4) (**A**) Ca^2+^-ATPase and (**B**) NA^+^-K^+^-ATPase. *P<0.05; **P<0.01; CCPR, conventional cardiopulmonary resuscitation; ECMO, extracorporeal membrane oxygenation; ECPR, extracorporeal cardiopulmonary resuscitation with ECMO strategies; ROSC, return of spontaneous circulation.

ECMO might also relieve brain injury by enhancing aerobic metabolism. Thus the Ca^2+^-ATPase and NA^+^-K^+^-ATPase activities in ECMO and CCPR groups were also compared. The results suggested that ECMO increased both Ca^2+^-ATPase and NA^+^-K^+^-ATPase activities compared with CCPR in pig brain after ventricular fibrillation (Figure 6).

### Brain histology and ultra-microstructure

**Figure 7:**
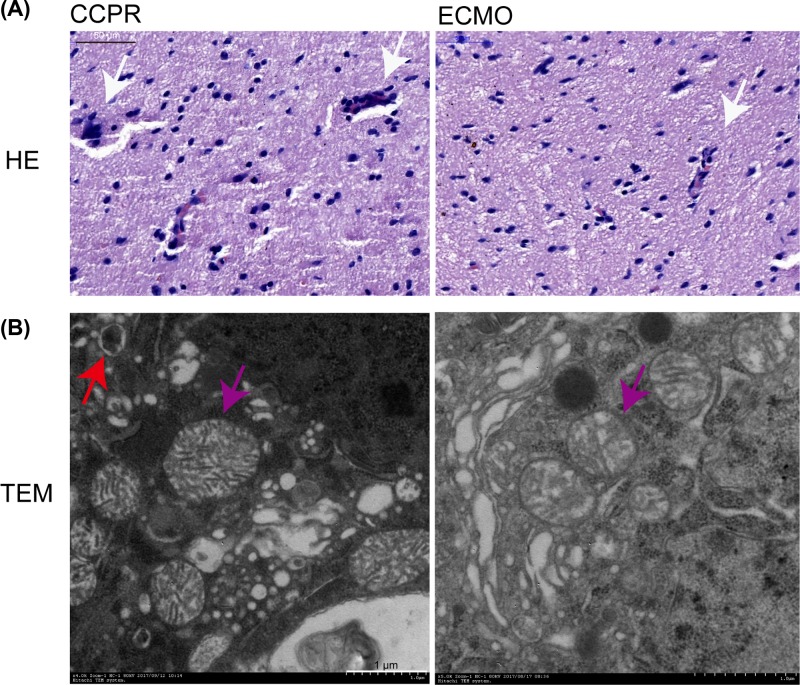
Representative images of hematoxylin and eosin staining and ultrastructure examination of the kidney tissues CA swine undergoing EPCR (*n*=8) or CCPR (*n*=4) (**A**) Hematoxylin and eosin staining. White arrow: infiltrated inflammatory cells. (**B**) Ultrastructure examination by TEM. Purple arrow: phagocytic vacuole; Red arrow: mitochondria. CCPR, conventional cardiopulmonary resuscitation; ECMO, extracorporeal membrane oxygenation; ECPR, extracorporeal cardiopulmonary resuscitation with ECMO strategies; ROSC, return of spontaneous circulation; TEM, transmission electron microscope.

Then brain tissues histology and ultra-microstructure in CCPR and ECMO groups were examined by hematoxylin and eosin staining and transmission electron microscope, respectively. In CCPR groups, the cells were found to present shrinkage, chromosome condensation, and nuclear pyknosis, and showed increased intercellular space and a large number of inflammatory cells were also found in CCPR group, while ECMO group showed an improved situation (Figure 7A). Under transmission electron microscope (TEM), the basement membranes were disrupted and mitochondria were swollen with disrupted cristae and phagocytic vacuole was increased in CCPR group, while the cells in ECMO groups had a relatively intact and clear structure (Figure 7B).

## Discussion

CA is a great health problem around the world [1,2]. ECMO, as an alternative resuscitative method, has been more increasingly and wildly used for refractory CA and improves survival rate and neurological outcomes in both OHCA and IHCA compared with CCPR [18]. The neurological outcomes are critical contributors to the mortality of CA patients. The underlying mechanisms how ECMO improves neurological outcomes of CA patients are not clear. In the present study, we compared the inflammatory response and ATPase activity in brain in swine model of CA between ECMO and CCPR treatments and found that pro-inflammatory cytokines IL-1, IL-6, TNFα, and TGFβ levels and Ca^2+^ - and NA^+^K^+^-ATPase activities were lower in ECMO group than CCRP group.

Many of the surviving CA patients develop severe neurological defect and other multi-organ dysfunction, also known as post-CA syndrome [27]. Evidence has shown that post-CA cerebral injury result in a death rate of 68% in OHCA and 23% in IHCA [28]. The underlying mechanism of post-resuscitation organ dysfunction may relate to inflammatory response in blood and tissues including brain [29]. Both animal experiments and clinical studies suggest that increased circulating pro-inflammatory cytokines such as IL-1β, IL-6, and TNFα in CA after successful CPR are associated with increased mortality [30–32]. Our previous *in vivo* studies also suggest that inhibition of TNFα levels by hypothermia contributes to the relief of neurological defect [33]. In the present study, we identified the significant decreased levels of IL-1, IL-6, and TNFα after ECMO compared with CCPR in CA swine but we did not observed the change of IL-10, a anti-inflammatory cytokine and protective factor during brain injury [26].

TGFβ is a super family of cytokines and plays a important role in cell growth, development, immunity, wound healing, inflammation, apoptosis, and cancer [34]. Currently, there are conflicting results about the effects of TGFβ in inflammation. A few reports suggested that TGFβ is a neuroprotective factor depending on the signaling pathways in different environmental conditions [35,36], however, more studies report that TGFβ can cause BBB disruption, hemorrhage, neuroinflammation, and cell death in various neurological diseases, indicating its role as excitotoxic and inflammatory factor [37–41]. Saghazadeh et al. [42] reveal that TGFβ can activate inflammatory cytokines expression including IL-1β and TNFα under several neurodegenerative conditions and Patel et al. [43] identify that traumatic brain injury-induced activation of TGFβ leads to neuroinflammation and neurodegeneration via phosphorylating Smad proteins in cultured rat cortical neurons. In our work, we observed the TGFβ was decreased in ECMO group compared with CCPR group. Combined with the IL-1 and TNFα results and the previous research, we hypothesized that TGFβ inhibition might attenuated brain injury partly via attenuating IL-1 and TNFα, which needed to be further validated in the future.

In our previous work, we observed that the increased activities of Ca^2+^-ATPase and NA^+^-K^+^-ATPase activities induced by drug treatment or therapeutic hypothermia could attenuate post-resuscitation organ dysfunction in swine model [33,44–47]. Here, we found the activities of Ca^2+^-ATPase and NA^+^-K^+^-ATPase in brain tissues in CA swine model were elevated by ECMO compared with CCPR.

Several limitations also exist in our study. We used the thermodilution method to measure CO, which might results in a slight but significant overestimation of CO [48,49] and might affect the examination of CO. However, we thought that this should not affect comparison of CO between ECMO group and CCPR group. And we have used large dose of heparin (5000 IU) to pre-coat circuit and membrane oxygenator during application of ECMO. The heparin, which was not used during CCPR, might also improve microcirculation and brain function in addition to ECMO [50]. We will clarify the effects of heparin on neurological outcomes during ECMO in future.

In summary, we preliminarily revealed the potential molecular mechanisms that ECMO improved neurological outcomes in CA animal model compared with CCPR partly via attenuating inflammation and increasing Ca^2+^-ATPase and NA^+^-K^+^-ATPase activities.

## Abbreviations

BBB: blood–brain barrier
CA: cardiac arrest
CCPR: conventional cardiopulmonary resuscitation
CO: cardiac output
CPR: cardiopulmonary resuscitation
ECG: electrocardiogram
ECMO: extracorporeal membrane oxygenation
ELISA: enzyme-linked immunosorbent assay
HR: heart rate
IHCA: in of hospital CA
MAP: mean arterial pressure
OHCA: out of hospital CA
ROSC: return of spontaneous circulation
TEM: transmission electron microscope
